# Do health systems delay the treatment of poor children? A qualitative study of child deaths in rural Tanzania

**DOI:** 10.1186/1472-6963-13-67

**Published:** 2013-02-19

**Authors:** Helle Samuelsen, Britt Pinkowski Tersbøl, Selemani Said Mbuyita

**Affiliations:** 1 Department of Anthropology, University of Copenhagen, Øster Farimagsgade 5, Copenhagen, DK 1353, Denmark; 2 Department of International Health, Immunology and Microbiology, University of Copenhagen, Copenhagen, Denmark; 3 Health System Thematic Group, Ifakara Health Institute, Ifakara, Tanzania

**Keywords:** Health systems, Delay, Child mortality, Tanzania, Treatment seeking, Doctor–patient relationship

## Abstract

**Background:**

Child mortality remains one of the major public-health problems in Tanzania. Delays in receiving and accessing adequate care contribute to these high rates. The literature on public health often focuses on the role of mothers in delaying treatment, suggesting that they contact the health system too late and that they prefer to treat their children at home, a perspective often echoed by health workers. Using the three-delay methodology, this study focus on the third phase of the model, exploring the delays experienced in receiving adequate care when mothers with a sick child contact a health-care facility. The overall objective is to analyse specific structural factors embedded in everyday practices at health facilities in a district in Tanzania which cause delays in the treatment of poor children and to discuss possible changes to institutions and social technologies.

**Methods:**

The study is based on qualitative fieldwork, including in-depth interviews with sixteen mothers who have lost a child, case studies in which patients were followed through the health system, and observations of more than a hundred consultations at all three levels of the health-care system. Data analysis took the form of thematic analysis.

**Results:**

Focusing on the third phase of the three-delay model, four main obstacles have been identified: confusions over payment, inadequate referral systems, the inefficient organization of health services and the culture of communication. These impediments strike the poorest segment of the mothers particularly hard. It is argued that these delaying factors function as ‘technologies of social exclusion’, as they are embedded in the everyday practices of the health facilities in systematic ways.

**Conclusion:**

The interviews, case studies and observations show that it is especially families with low social and cultural capital that experience delays after having contacted the health-care system. Reductions of the various types of uncertainty concerning payment, improved referral practices and improved communication between health staff and patients would reduce some of the delays within health facilities, which might feedback positively into the other two phases of delay.

## Background

Child mortality is one of the major public-health challenges in Tanzania, as well as in other low-income countries. With an under-five mortality rate of 81 per 1,000, [1] child mortality remains a public health concern. Recent DHS studies show that levels of infant and child mortality are highest among women with no education and that mortality levels are associated with wealth quintiles [1,2]. Poor nutrition and hygiene, as well as lack of access to social support, are among the most important reasons [3,4]. Delay in accessing and receiving adequate care contributes to the high mortality rates, and there has been a strong focus on the role of distance and cost. Thaddeus and Maine [5] have put forward the three-delay model as a framework for analysing factors affecting the interval between the onset of symptoms and the outcome of the illness. The first phase of delay takes place at home, where there might be a delay in deciding to seek care on the part of the individual, the family or both (p. 1099). The second phase concerns delay in reaching an adequate health-care facility, the third phase delay in receiving adequate care at the facility. The three-delay model was originally developed in relation to studies on maternal mortality, where the focus on the third phase was particularly emphasised. In contrast to this, studies of delays relating to child mortality have most often focused on the first phase of delay at the household level. The delay occurring here has been ascribed to uncertainty about the seriousness of the child’s condition and preference for home-based treatment with biomedical drugs or local herbs [6]. Alternatively mothers choose to consult traditional healers first [7]. There is much to be gained from addressing all phases of delay described by Thaddeus and Maine and from acknowledging that the various types of delays interact with each other [5,8,9]. However, this article is based on a study of the third phase of delay, that is, delays at biomedical health-care facilities. The overall aim of the article is to analyse the specific structural factors that cause delays in the treatment of children. From the patient’s perspective – or rather the perspective of the parent of a sick child – we look at processes of delays in reaching adequate health care after contacting a health facility.

We use the notion of *biomedical technologies* in our analysis of delays at health facilities. Following Rose, *biomedical technologies* are assemblages of material objects, knowledge, persons and systems of judgements, which in their practice are culturally framed by certain presuppositions and assumptions about human beings [10]. In addition, we discuss what changes could be adopted to reduce these delays. In this discussion, we apply the ‘power, trust, risk nexus’ as described by Grimen [11] as an underlying conceptual framework for the study. This conceptual framework takes the notion of power into consideration in the sense that the health professional–patient relationship is characterised by a hierarchy in which the former is superior and the latter by definition inferior. This is due to a knowledge gap, with the health professional being expected to have superior knowledge about disease and treatment, and the patient needing to have trust in the health professional without any guarantee that no harm will occur.

## Method

### Study setting

Fieldwork for this study was conducted in Mpwapwa District in the Dodoma Region of central Tanzania. Mpwapwa District has a population of about a quarter of a million [12]. Child mortality levels in the district stagnated during the period from 1988 to 2002, with an under-five mortality rate in 2002 of 217 per 1000 births. The majority of the population is Wagogo, with a minority of Hehe living in the northern part of the district. These were traditionally sedentary but mobile cultivators subsisting on sorghum and millet, but also with a considerable number of livestock [13]. Today, the population of this district is occasionally provided with food aid, as long periods of drought make it difficult to subsist on farming alone. In Tanzania, health services are offered at three levels. The dispensary is the unit at which the most basic services are provided. It is run by a clinical officer (two years of education) and normally serves a population of about 10,000. The health centre forms the secondary level of the government health system. It is equipped with simple diagnostic and treatment facilities and a small in-patient department and serves about 50,000 people. The third level consists of the hospitals (district, regional and university hospitals). Each of the 126 districts in the country is supposed to have a district hospital covering a population of about 250,000 [14]. Most of the health staff have been trained in IMCI (Integrated Management of Childhood Illnesses), a strategy to reduce child deaths and the frequency and severity of child illnesses and disability. IMCI includes interventions to improve health workers’ case-management and counselling practices.

### Data collection

Fieldwork for this article was carried out in 2007. We had been involved in a multidisciplinary study conducted on behalf of the Ministry of Health and Danida the year before, focusing on differences in child mortality in four districts of Tanzania. In that study, the district of Mpwapwa was defined as an ‘underperformer’ (with a lower decrease in child mortality than expected) based on a comparison of census data from 1988 and 2002 adjusted for non-health system-related factors [15]. In 2007 we had an opportunity to conduct a follow-up study where we were interested in focusing in more detail on concrete experiences with child deaths and access for the treatment of sick children. The fieldwork in 2007 included a total of sixteen mothers who had lost a child two months to one year earlier (see Table 1 for an overview of mothers’ accounts). The mothers were recruited from three different villages: one with a dispensary, one with a health-care centre and one close to the district hospital. Villages with a health facility were purposely selected in order to eliminate long distance as a barrier to contact with the primary-level health-care system. In each of the three selected villages, the village head was contacted in order to ask permission to conduct interviews in the village and to help us identify women who had lost a child during the specified period. We also conducted participant observation – or rather ‘negotiated interactive observations’, to use Wind’s terminology– at the health facilities by following the children and their mothers through the facility from registration to admission or discharge [16]. The cases followed through the facility were randomly selected at the point of registration. We followed them until they were admitted and the child had received treatment, or alternatively until the child had obtained medication and was heading home with the mother. In addition, we observed about a hundred consultations at the out-patients department (OPD) and mother-child-health (MCH) services at all three levels of care in the district.

**Table 1:** Mothers who lost a child

**Name of mother**	**Age of child**	**Symptoms**	**Treatment-seeking actions**	**Outcome**
Leah	1 year	Sudden vomiting, swelling stomach	Went to dispensary same day, and later to district hospital	The child died the following day at the hospital
Happiness	5 months	Fever, convulsions/*degedege*	First treatment with medicines at home, then went to health centre	The child died on the sixth day at the health centre
Dorcas	New-born	Difficulties with the umbilical cord. The mother thinks the nurse fixed the cord wrongly.	Delivery took place at health centre	The child started to suck, but died while mother was asleep. Died at health centre.
Esther	2 years 9 months	Stomach problems for four months: frequent diarrhoea with foam and/or blood. Anaemia.	First treatment with herbs at home. Then they went directly to district hospital.	Three days at hospital with drops, ORS and medicine. Blood transfusion was planned, but the child died at the hospital.
Grace	1 year 11 months	From birth the child was often sick, body and eyes became greenish, and the child had fever several times. The child was anaemic.	Went to district hospital four times to get treatment (medicine and blood transfusion).	The child died at hospital two weeks after admission.
Agnes	2 years	Started with fever and anaemia; later the whole body swelled and bad smelling water came out of the nose. The child was sick for four months.	Was admitted to district hospital in the beginning. Later tried traditional healer. In the last phase, the child was prescribed injections by the dispensary.	The child died at home without finishing all the prescribed injections at the dispensary.
Hogra	5 months	Started with vomiting and diarrhoea.	Went to the dispensary, where the child was prescribed five injections at five-hour intervals.	The child died at home on the second day.
Maria M	New-born	The child was delivered by caesarean section. The mother does not know of any symptoms.	The mother delivered at the health centre.	The child died about twelve hours after birth.
Agnes P	1 year	Fever and *degedege.*	Went to dispensary, where medicine was prescribed. Went again next day and third day she went to district hospital, where the child was admitted with drops and medicine.	The child died at the hospital on the third day of the sickness.
Maria K	5 months	Fever and *degedege*	Went to health centre, where the child was admitted and treated for two days. After one day, the child was admitted again.	The child died at the health centre after three days of sickness.
Lucy	1 year 2 months	Vomiting and diarrhoea, and fever. Dehydration.	Went to dispensary, but clinical officer was drunk and did not receive them. Bought medicine at drug store. Next morning went to district hospital, where the child was admitted and treated.	The child died at the hospital on the day of admission.
Hawa	5 years	Had episodes of chest problems since birth. Coughing started and the whole body started to swell.	Went to dispensary and hospital during some episodes. Went to mission hospital and was referred to Muhimbili. Was referred to India for operation. Had also consulted traditional healer who referred child to hospital.	The child was diagnosed with heart problems (hole in the heart). The child died at home.
Amina	4 months	Fever and later *degedege.*	Went to dispensary for treatment, consulted traditional healer for the *degedege.* Was referred to district hospital.	The child died during the first night at hospital.
Janet	9 months	The child’s head started to swell, and eventually the child did not want to breastfeed.	Went to the district hospital. Were referred to Muhimbili, but due to financial constraints they did not go.	The child died at home after five months.
Velian	6 months	Fever, coughing and breathing problems.	Went to the dispensary for treatment.	The child died at home.
Melusela	4 months	Fever, whining and not willing to breastfeed.	Did not consult health facilities.	The child died at home after three days.

Summary of mothers’ accounts.

### Ethics

Research permission and ethical clearance were obtained at COSTECH (the Tanzania Commission of Science and Technology), and both the District Executive Director and the District Medical Officer were informed about our study and debriefed at the end of the fieldwork. The study was carried out in full compliance with the Helsinki Declaration. The interviewees all gave informed consent and were asked to sign a consent form. Interviewees were informed about the study and the meaning of confidentiality and anonymity. During the informed consent process, it was made clear what we aimed to talk about and why, and we specifically asked each woman if she would be comfortable discussing this highly sensitive topic. The in-depth interviews were conducted by the authors with translators and focused on the mother’s recall of illness and health-seeking events that occurred prior to their child’s death. Great care was taken to develop trusting and open communication and to ensure that the interviewees felt comfortable with the interview situation. The location for each of the interviews was carefully selected in consultation with the women to ensure privacy, and we also made the women aware that they could withdraw from the interview at any point. However, none of the mothers were hesitant in discussing the interview topic. Instead, they appeared to appreciate our interest and the opportunity to relate their experiences. The atmosphere was generally relaxed, and the mothers appeared to feel free to share their experiences.

With respect to the negotiated interactive observations in the consultation room and when following cases through the health facilities, it was expected that health-care professionals might change their behaviour due to the presence of researchers in the consultation room. However, after a few consultations, the health-care staff were also observed to be relaxed and returned to carrying out consultations the way they are used to doing. This was confirmed by the patients when they were asked to compare the observed consultation to previous consultations they have experienced; and in their view, the observed consultations resembled earlier consultation experiences. We also asked informants about the consultation procedures they experienced during those consultations we did not observe. Their account of the consultation clearly resembled the observed consultations.

During the process of following cases through the facility, we did not always remain neutral observers. In a few cases in which we encountered seriously ill children, we offered transportation to the hospital or intervened to shorten waiting times, and in a few cases we paid for medicine at a private pharmacy to avoid a delay of treatment of several days. These interventions did, of course, have an impact on the course of treatment in these few cases, but the fact that we had to intervene for ethical reasons illustrates the vulnerability of some families seeking health care.

### Data handling

The authors were given permission to tape-record interviews (except in one case, when extensive field notes were taken instead), and the recordings were transcribed. Quality checks of the transcriptions were carried out by an independent research assistant. Detailed notes were taken during observations of consultations and when following cases of illness through the system. All the interview material was read by the authors, and common themes emerging from the interviews were identified. Themes and codes were arrived at inductively from the transcribed interviews and compared and contrasted to the notes from observations. Dialogues from the consultation room are quoted in this article where they constitute typical examples of consultations either because of their short duration or because they represent a typical style of communication between health professionals and mothers. The four obstacles described in our results section came out as common themes through the content analysis of different types of data (interview transcripts and notes of consultations and of the cases followed through the health facility).

## Results

This study includes interviews with sixteen women who had lost a child within the previous two years, observation of approximately a hundred OPD consultations with children, and participatory observation of the route and process that mothers were required to go through with their children at health facilities, as well as interactions between the mothers and health-care professionals. The women we interviewed were between 17 and 43 years old. A few of the women had never attended school; one had completed secondary school, while the rest had between four and seven years of schooling. Half of the women (eight) were not married (three were widows). Most of the households lived from subsistence farming, whereas three women’s husbands had some kind of wage income (a policeman, a watchman and a businessman). One woman did not consult a health facility during the child’s sickness. The more than one hundred OPD consultations observed generally included mothers with young children (presenting with fever, coughs and diarrhoea as the main symptoms) and were of very short duration (2–6 minutes).

We identify four major obstacles to timely and adequate access to bio-medical care: confusions over payment, inadequate referral systems, inefficient organization of health services and communication problems between health professionals and patients.

The first obstacle that causes treatment delay has to do with payment. Despite the Tanzanian exemption policy, which should allow all treatment (consultation and medicine) of children younger than five years to be free, parents are often required to pay for medicines and treatment at the health facility. However, as payments are not fixed but occasional and unpredictable, they might cause delays as the mother first has to provide the money before treatment is initiated. Another important barrier causing delay is that many children are not successfully referred to someone with the required medical expertise. The third obstacle delaying adequate care has to do with the ways in which the health services are organised within the health facility: often the sick child and members of its family are instructed to go to several points in and outside the facility before treatment is obtained. Finally, we identify communicative obstacles, with the health staff consistently using specific forms of interaction which compromise the least educated and poorest families. In the following sections, we present the four main obstacles identified across all data types.

### Confusions over payment

The following example illustrates the first obstacle concerning confusions over payment.

Agnes is thirty years old and lives with her husband and child in a village in Mpwapwa District. Agnes has already lost four children, two of whom died in hospital and two at home. The sickness of the last child started three months before it died. The child was two years old and, according to Agnes, initially suffered from *homa* (fever) and anaemia (see Table 1). She took the child to the district hospital, where it received a blood transfusion, with its father as the donor. A week after admission, the child was all right again and was discharged, but after a month its body started to swell, skin sores appeared and its skin started peeling off. Agnes consulted a traditional healer, but the treatment was not a success. Then she went to the nearest dispensary, where she was given some medicine and cream for the sores. However, the child did not improve, so they went back to the dispensary, where it was given a number of quinine injections. There were no convulsions (*degedege*). They paid 5,000 Tanzania Shillings (Tsh) to the *mganga* (traditional healer) and a total of Tsh 10,000 for the two visits to the dispensary. The payment was made directly to the clinical officer, and they were told it was for medicine. The husband had to borrow money in order to pay the Tsh 10,000, and the parents still needed to pay Tsh 2,000 back to people who had lent them money. They were not referred to more advanced care at the District Hospital. Subsequently, the child died at home. Amina and her husband were asked to pay 15,000 Tanzania Shillings (Tsh) for medicine at the dispensary when bringing their four months old feverish baby for treatment. The husband went home to sell a few sacks of maize in order to get enough cash. Amina says: ‘while we were at Mpwapwa, our money finished so my husband had to go back home for some more money’. During the illness period, they also consulted a traditional healer. He only demanded 100 Tanzania Shillings as his treatment did not work. The traditional healer advised them to contact the District Hospital. They were not asked for payment at the District Hospital. Maria K had to pay 2,800 Tanzania Shillings for medicine at health centre for the treatment of her five months old baby suffering from fever and *degedege*. She was accompanied to the health centre by her mother, who managed to borrow the money from acquaintances in the villages. These cases, where family members have to return to the village in order to borrow money for the treatment of the acutely ill children often imply treatment delays of several hours.

### Inadequate referral systems

Mothers may be asked to take their children to a higher level of the health system, but they have to organise transport on their own. The district has one or two functioning ambulances, but since money for fuel is short, they are rarely used. Registration data from 2000–2006 show that Mpwapwa referred significantly fewer children to the district hospital compared to Kondoa, the neighbouring district. Furthermore, in Mpwapwa there was a clear tendency only to refer children with burns or severe injuries: children with severe malaria or other infections were rarely referred (National Institute of Medical Research IHRC, CISU: Assessment of Child Mortality in Selected Districts of Tanzania, unpublished, p.23).

In the case of Agnes, the clinical officer did not refer the severely ill child to a higher level of the health-care system. This was also the case for Happiness, a 26-year-old woman who lost her two children (see Table 1). The first child was stillborn, and Happiness was told that her second child died of cerebral malaria. The small boy started to develop a high fever: ‘I thought it was a normal fever and I was giving him medicine at home’, Happiness told us. She gave him Paracetamol, Panadol and Septrin syrup. However, the child’s condition worsened, so she consulted the village dispensary, where the nurse advised her to take the child to the government health centre because it seemed to be dehydrated and anaemic. At the health centre the child was examined for dehydration and anaemia, but the test results showed neither. The child developed convulsions and was admitted to the health centre, where they dispensed quinine drips. Its condition improved on the first day, but worsened on the second day. The health staff again prescribed injections. Happiness says: “At that time, the baby could not suck; whatever you gave him, came back’. ‘ […] On the third day, the baby’s condition did not change. I could not give him medicine because whatever I gave, he was vomiting. The baby was just *kukema* (an unusual sound of a child who is attacked by pneumonia). It persisted until the baby died on the sixth day.’ The child was admitted to the health centre and remained there until his death on the sixth day. We asked Happiness whether the health centre had talked to her about referring them to higher level care at the district hospital. She said: ‘They did not mention such a thing. They kept on prescribing some medicines while the child did not want to swallow.’ Happiness paid a total of about Tsh 10,000 for medicines, syringes and needles. Lucy and her husband had to refer themselves and their child suffering from severe diarrhoea and vomiting to the District Hospital after having knocked on the door of the dispensary for five hours late one evening. The clinical officer was drunk and did not open the door. They then managed to wake up the local pharmacist who provided them with medication. However, the child’s condition did not improve, so Lucy and her husband set out early next morning on a four hours bike ride with the sick child in order to reach the district hospital. The child was admitted and treated at the hospital, but died on the day of admission. It may of course be difficult to judge whether the the child ought to have been referred to a higher level of the health system, since we only hear the mothers’ side of the story. In Hogra’s case for example, it might be difficult to say whether the dispensary delayed adequate treatment. The child was suffering from diarrhoea and vomiting when Hogra contacted the dispensary. A total of five injections with a five-hour interval were prescribed. The child was not admitted; Hogra took the child to the dispensary for the injections. The child died the following day. However, the general picture from our case studies is that dispensaries and health centres only in rare cases refer the acutely ill children to the district hospital.

### Inefficient organization of health services

Data from observations of out-patient care for children at the dispensary, health centre and hospital levels suggest that there are several obstacles to the prompt treatment of seriously ill children. The issue of absenteeism is crucial. Mothers seeking help from a health facility may find that the dispensary or health centre has no qualified staff for a period of time. This may be due to training activities, annual leave or illness. The research team visited a health centre with a permanent staff of about fifteen health professionals. Over three consecutive days, the highest ranking member of staff was an assistant dental officer, who was responsible for the ten to fifteen patients admitted as well as the OPD services (with some thirty to forty consultations a day). The assistant medical officer, two clinical officers and a number of nurses were all absent for various reasons.

No triage appears to be performed to ensure fast examination and treatment of the most severely ill children at any of the observed facilities. Often a first come first served principle rules, but on one occasion we also observed a clinical officer come out of the consultation room, collect all the registration booklets (the patient’s health file) and call in patients in an arbitrary order.

Observations at district hospitals show that carers with seriously ill children have to move around extensively inside and outside the hospital premises before the child receives treatment. One young couple arrived at the district hospital one morning at around 11 am. Their child was suffering from fever and breathing very rapidly. After the registration and consultation at the OPD, they were sent to the laboratory for a malaria test. Then they went back to the clinical officer at the OPD and were told that their child would be admitted. Then they had to go to the mother-child-health clinic for medication, gloves and a tube for the blood transfusion and to the hospital pharmacy to obtain other medicine. Arriving at the ward, the nurses sent them for a blood test in order to identify a donor for a blood transfusion for the child. It was determined that the mother could donate blood. She also had an HIV and a hepatitis test. The parents were then sent back to the ward, where the assistant medical officer again asked the husband to obtain additional medicine from the hospital pharmacy, where he was made to wait in a queue. Returning with the medicine, he was now asked to go out and buy some clean drinking water for spoon-feeding the child. Returning to the ward, where the mother and child had now been allocated a bed, the AMO (Assistant Medical Officer) called the MCH to ask for another type of medicine, which the district hospital did not have in stock. The father was sent to a private pharmacy outside the hospital to obtain the medicine, which he had to pay for. At this point, the child had still not received any treatment. While we acknowledge that diagnosis and identification of the correct therapy is a process, it is also clear that its organisation may significantly delay treatment. Furthermore, it confuses the parents. Wishing to show respect to the health authorities, and uncertain about their obligations and the actual procedures at the health facilities, the parents scramble around the facility and its surroundings.

### Communicative practices

Every morning, the waiting area at the outpatients department in the district hospital at Mpwapwa is crowded with people, especially when the malaria season peaks. One morning in October 2007, we observed the first eighteen consultations carried out by one of the clinical officers. Most of them were very short, as the verbatim translation below illustrates:

CO: ‘What is the problem with the child?’

Mother: ‘The child is suffering from flu and coughing’.

CO: ‘Does the child breastfeed properly?’

Mother: ‘No, she doesn’t’.

CO: ‘I am prescribing the child medicine, and when you arrive home you have to make sure you give her this. Is that OK?’

Mother: ‘Yes’.

This case is a good example of magic in medicine, as described by van der Geest: ‘Writing a prescription can best be described as a closing ritual which is intended – and often succeeds – to send the patient away with hope and positive feelings towards his medical problem, himself and the doctor’ [17]. The positive appreciation of the prescription may conceal both the uncertainty that still exists and the ‘patient’s disappointment about the shortness of the encounter’ [17:140]. The agency of the doctor (or in more general terms the health provider) is extremely important to the patient. The prescription or the clinical officer’s instruction to get a test at the laboratory is appreciated by the patients and seen as a symbol of the doctor’s professional skills and authority. Only one out of the sixteen mothers interviewed, however, recall having received any information about the diagnosis and cause of the child’s illness. The one exception is the case of Howa, whose child was referred to Muhimbili hospital with a heart failure.

However, during the twelfth consultation that morning, the CO seems to be somewhat irritated (the quotes are ad verbatim):

CO: ‘How are you, mother?’

Mother: ‘Fine, but my child does not feel well’.

CO: ‘What’s your child’s problem?’

Mother: ‘The child is sick; she has stomachache and headache’

CO: ‘How old is your child?’

Mother: ‘She is two years old’

CO: ‘Do you boil the drinking water?’

Mother: ‘No’

CO: ‘Why? Don’t you know the effects of drinking unboiled water?’

Mother: ‘No’

CO: ‘Your family may get diarrhoea, typhoid etc. So you should boil the drinking water. Do you understand me?’

Mother: ‘Yes, I understand’

CO: ‘Does your child have any other problem?’

Mother: ‘No’

CO: ‘I saw the child was coughing before, wasn’t she?’

Mother: ‘Yes, she is coughing’

CO: ‘Don’t you know coughing is a problem?’

No answer from the mother

CO: ‘Does she breathe very quickly?’

Mother: ‘No’

CO: ‘When did she start the illness?’

Mother: ‘About one month and a half ago’

CO: ‘Does she get diarrhoea?’

Mother: ‘Yes’

CO: ‘Do the child’s legs become big?’

Mother: ‘Yes’

CO: ‘Does she eat properly?’

Mother: ‘No, she doesn’t, she always chooses the foods. When I gave her beans and soup she got diarrhoea, and when I gave her some milk she vomited’

CO: ‘Does she get a problem when she urinates?’

Mother: ‘No’

CO: ‘Do you use a mosquito net in your home?’

Mother: ‘No’

CO: ‘Why not?’

Mother: ‘There are no mosquitoes’

CO: ‘Go to the laboratory and test the child’s blood. Your child is suffering from malnutrition. So you have to make sure your child is getting a balanced diet. Do you understand?’

Mother: ‘Yes, I do.’

[The mother leaves the room in order to join the queue at the laboratory].

This case shows that, although the clinical officer is very observant, noticing a cough, the mother is spoken to in a somewhat patronising and arrogant manner. In some cases, the patients take on the guilt and feel that the scolding by the health staff is justified. Esther (see Table 1), who lost her two-year-old son while she was pregnant with her next child, says: ‘I was not embarrassed; they had a right to be furious because they wanted to help the child’, explaining further: ‘It was because there were some negligence and poverty on our side’. We see this statement as an expression of Grimen’s ‘power, trust risk nexus’. Esther acknowledges the asymmetric relation between herself and the health professionals. She takes on the blame for the death of her child while she from her structurally inferior position, trusts that the health professionals potentially had the skills and knowledge to save her child.

Parents may at times also be met with various demands from service providers which underline the inferior status of patients and add to the unpredictability of seeking care. We have observed parents being asked to fetch fruit juice for an admitted child to drink with the prescribed drugs, and a mother being asked to pay for tea for a provider who felt tired and refused to begin the consultation until he had received it. In another instance, an assistant medical officer reprimanded a father for not having dressed his extremely ill child properly before rushing to the hospital, and he refused to treat the child until it had been dressed.

## Discussion

### Study limitations

We would have liked to be able to triangulate mothers’ accounts of the route to and timeframe for seeking health care with other data. Participant observation may not have been appropriate due to the ethical concerns. Prolonged periods of participant observation in the communities and households of informants would have allowed for better contextualisation of mother’ accounts of the events leading up to the death of their children. However, given that several other scholars have focused on the community level to ascertain health-seeking practices and the decision-making processes that underlie them, [6,18-21] in this study we prioritised exploring the constraints and delay experienced by mothers and other carers at the health facilities.

### Reduction of uncertainties

This study has focused on the delays in receiving adequate care at health facilities (the third delay) and describes four factors that particularly affect poor families: confusion over payment, inadequate referral practices, inefficient organisation within the health facility and communication difficulties. These obstacles seem to be difficult to overcome for people with low cultural and social capital and constitute a silent form of structural violence, which hits the poorest people hardest [22]. The structural inferiority on the part of the patient is as suggested by Grimen partly determined by the knowledge gap between the health professional and the patient [11]. In addition, patients are by definition in a vulnerable position due either to their own physical or mental illness or to the stress and worries of having a sick child. In our study, health-workers often blamed parents for delays in seeking health care and maintained that this problem remains one of the major challenges in reducing child mortality in their district. In contrast to this, we found that significant delays take place while parents are actually seeking care (between different levels of formal health care), not least at the health facilities themselves, while parents (mothers) attempt to accommodate health professionals’ demands. As Fassin has pointed out, health professionals refer to ‘cultural beliefs’ as the reason why women appear to avoid the bio-medical health system: ‘In incriminating culture, as certain health authorities willingly do, sometimes supported by anthropological data, they are in fact blaming victims whilst masking their own responsibility in the matter’ [23]. Anthropologists may have contributed to this discussion with detailed empirical studies of local perceptions of illness and how these influence biomedical treatment [6,18-20,24-26]. While these studies are of course justified in themselves, they might have contributed to the strong focus on the first type of delay. The structural inferiority of the patient is, as mentioned, partly determined by the knowledge gap between the health professional and the patients [11]. Patients are by definition in a vulnerable position due either to their own physical or mental illnesses or to the stress and worries of having a sick child.

Schellenberg’s study of rural Tanzania shows that the rate of hospital admissions for the lowest socioeconomic status quintile was almost half that of the highest, despite the fact that there was no significant connection between socioeconomic status and the reported prevalence of fever, diarrhoea, severe diarrhoea or pneumonia [27]. In an editorial for *Tropical Medicine and International Health*, Lambert and Van der Stuyft, discussing delays to tuberculosis treatment, ask whether it is fair to continue to blame the patient when problems in the health service such as the accessibility or quality of services can also contribute to patient delay [28]. However, the suboptimum working conditions of health workers also play a role here [29].

According to our findings, patients are not ignorant of the symptoms of serious illnesses, nor do they refuse to seek biomedical therapy, but they meet a number of obstacles during the treatment seeking process. These obstacles are connected to the organization of health services, as well as to social interactions between health professionals and patients. The problem of the inadequate referral system has been pointed out in other studies. Walter et al. found that health workers disagreed with the IMCI guide that all seriously ill children should be referred [30]. In their study, among 81 health workers who were interviewed, 68% responded that severe malaria could be managed without a referral, and only 5% reported ever having withheld referral because the child’s condition appeared hopeless [30]. The lack of any type of triage at the district hospital may perhaps not be very different from the situation in an out-patients department at a European hospital, but the fact that malaria can kill a child within 24 hours of the onset of symptoms makes it crucial to perform triage among the many patients in the crowded patients’ department and to act promptly if a severely ill child is identified.

We call the four impediments elaborated here *technologies of social exclusion*, as they all disfavour the patients with the least social and cultural capital. Through exclusion or ‘technologies of invisibility’, as Biehl shows in an analysis of AIDS treatment in Brazil [31], the most marginal population groups become ‘absent things’.

## Conclusion

Using the three-delay framework proposed by Thaddeus and Maine [5], in this study we have primarily focused on the third delay, that in receiving adequate care. Our cases, interviews and observations show that many rural families experience delays after having contacted the health-care system. They face problems affording the medicines, thus contributing to the delaying of treatment. They do not immediately catch the meaning of the instructions provided by the health professionals during the three- to five-minute consultations. They do not dress their children appropriately, which is interpreted by health-care staff as showing a lack of respect for their services. With globalisation, the rural poor with little or no schooling are increasingly becoming marginalised, and their social competencies in how to ‘behave’ in and ‘navigate’ through bureaucratic systems such as the health-care system are increasingly being exposed, leaving them behind as ‘redundant’[32]. The technologies of the health institutions constantly remind the 'incompetent patients’ of their inferior position. While it is well known that poor people are also disadvantaged in relation to their access to quality health care, it is particularly worrying that the forms of *positional suffering*[33] described here are being reproduced in a public-health system that has an obligation to implement a pro-poor health policy. As Paul Farmer has suggested, ‘poor people are not only more likely to suffer, they are also more likely to have their suffering silenced’ [34].

The three phases of delay are, of course, interrelated. The barriers in accessing the health-care system and the inadequate care received at phases 2 and. 3 feed back into phase 1, where delays in decision-making may increase. So, how can improvements to the organization of services optimize patient treatment?

First at the country level, the government must determine whether it is realistic to provide free services and drugs for children under five: if not, clear guidelines as to when and for what parents must pay should be provided. Uncertainty about payment is particularly a problem for people living on the margins.

Time could be saved by reorganising and coordinating the reception, admission and treatment procedures for severely ill children. Some of these improvements might be cost-free, such as ensuring that carers waiting in line at the OPD are screened and examined swiftly and assisted through the admissions process.

Furthermore, the important issue of absenteeism must be addressed [35]. It is clear that sustainable solutions to improved quality of services demand good and strong leadership and accountability, both at the district level and in the individual facilities. Another issue that should be addressed is how IMCI can be efficiently implemented through training and continuous supervision. In a multi-country study, Huicho et al. found that in Tanzania those with a longer period of pre-service training performed better in the integrated assessment of sick children than those with a shorter period [36]. This may encourage more parents to choose the public-health system as a first resort if the facilities are able to reduce unpredictability and increase trust, as well as in situations of extreme stress, such as when a child is seriously ill.

## Abbreviations

AMO: Assistant medical officer; CO: Clinical officer; HIV: Human immunodefect virus; IMCI: Integrated management of childhood illnesses; MCH: Mother child health; OPD: Out patient department; Tsh: Tanzania shillings.

## Competing interests

The authors declare that they have no competing interests.

## Authors’ contribution

HS planned and wrote the protocol, collected and analysed data, and was the lead author of the manuscript. BPT contributed substantially to the protocol development and data collection and contributed to the writing of the manuscript. SSM participated in data collection and contributed to the writing of the manuscript. All authors have read and approved the final version.

## Pre-publication history

The pre-publication history for this paper can be accessed here:

http://www.biomedcentral.com/1472-6963/13/67/prepub
